# Reframing Aging and Diabetes: Centering Transgender Health Equity

**DOI:** 10.1093/geroni/igaf122.2405

**Published:** 2025-12-31

**Authors:** Barbara Mendez Campos, Gabi Celia Ortiz

**Affiliations:** University of Tennessee Knoxville, Knoxville, Tennessee, United States; Boston College, Chestnut Hill, Massachusetts, United States

## Abstract

As more transgender (trans) individuals reach older adulthood, it is essential to understand their health risks, including chronic conditions such as diabetes. Research on aging and diabetes has largely excluded trans populations, leaving gaps in knowledge about their unique experiences in healthcare access, diagnosis, and management. This study utilizes pooled data from the 2021–2023 California Health Interview Survey (CHIS) to examine predictors of prediabetes and diabetes among trans individuals 18 years and older (n = 322, weighted N = 249,822). Logistic regression, accounting for survey weights and jackknife estimation, found that adults aged 80+, those with high blood pressure, those with higher education, and those with health insurance had statistically significantly higher odds of diabetes. Findings suggest that both structural and individual factors contribute to prediabetes and diabetes risk among trans adults, with critical implications for aging. The association between health insurance and diagnosis highlights a link to healthcare access as a key aspect in diabetes detection, raising concerns about undiagnosed cases among uninsured trans individuals. Similarly, the protective effect of education may reflect greater health literacy, yet disparities in education and healthcare access persist, limiting prevention opportunities. Larger samples are needed to refine estimates, assess racial and ethnic differences, and explore key interactions shaping diabetes risk. Through centering trans adults, findings underscore the need for public health initiatives that bridge healthcare gaps and ensure culturally competent and gender-relevant diabetes prevention and management. Broadly, they emphasize the importance of including trans experiences in aging research, healthcare, and services to advance equitable health outcomes.

